# High-Quality Draft Genome Sequences of *Xanthomonas axonopodis* pv. glycines Strains CFBP 2526 and CFBP 7119

**DOI:** 10.1128/genomeA.01036-13

**Published:** 2013-12-12

**Authors:** A. Darrasse, S. Bolot, L. Serres-Giardi, E. Charbit, T. Boureau, M. Fisher-Le Saux, M. Briand, M. Arlat, L. Gagnevin, R. Koebnik, L. D. Noël, S. Carrère, M. A. Jacques

**Affiliations:** INRA, Institut de Recherche en Horticulture et Semences (IRHS), UMR 1345 SFR 4207 QUASAV, Beaucouzé, Francea; Université d’Angers, Institut de Recherche en Horticulture et Semences (IRHS), UMR 1345 SFR 4207 QUASAV, Beaucouzé, Franceb; Agrocampus Ouest, Institut de Recherche en Horticulture et Semences (IRHS), UMR 1345 SFR 4207 QUASAV, Beaucouzé, Francec; INRA, Laboratoire des Interactions Plantes Micro-Organismes (LIPM), UMR 441, Castanet-Tolosan, Franced; CNRS, Laboratoire des Interactions Plantes Micro-Organismes (LIPM), UMR 2594, Castanet-Tolosan, Francee; Université de Toulouse, Université Paul Sabatier, Toulouse, Francef; UMR “Peuplements Végétaux et Bioagresseurs en Milieu Tropical” (PVBMT), CIRAD, Saint-Pierre, La Réunion, Franceg; UMR 186 IRD-Cirad-Université Montpellier 2 “Résistance des Plantes aux Bioaggresseurs,” Montpellier, Franceh

## Abstract

We report here the high-quality draft genome sequences of two strains of *Xanthomonas axonopodis* pv. glycines, the causal agent of bacterial pustule on soybeans. Comparison of these genomes with those of phylogenetically closely related pathovars of *Xanthomonas* spp. will help to understand the mechanisms involved in host specificity and adaptation to host plants.

## GENOME ANNOUNCEMENT

The soybean (*Glycine max*) is an economically important crop legume for seed proteins and oil content (1), particularly in America and Asia (2). *Xanthomonas axonopodis* pv. glycines causes the bacterial pustule on soybeans. Typical symptoms are pale green spots with elevated pustules due to the hypertrophy of parenchyma cells (3). Expanding lesions become necrotic and cause premature defoliation. Symptoms may develop on pods, and the pathogen is transmitted via the seeds. The disease reduces yield and crop quality and occurs during warm and wet weather (4).

A single recessive locus, *rxp*, which carries resistance to bacterial pustules, has been identified (5) and is present in the resistant cultivar Williams 82, whose genome is sequenced (6). Several studies on the diversity of *Xanthomonas axonopodis* pv. glycines described at least 3 races (7, 8) according to the distribution of transcription activator-like (TAL) type III effectors, which are major pathogenicity factors of *Xanthomonas axonopodis* pv. glycines on soybeans (9–11). Several genes involved in various infection processes are controlled by two major regulators (12, 13). The genome of *Xanthomonas axonopodis* pv. glycines strain 12-2, isolated in Thailand, was recently published (12). We present the genomes of two *Xanthomonas axonopodis* pv. glycines strains, CFBP 2526 and CFBP 7119, isolated in Sudan and Brazil, respectively. These strains were included in several phylogenetic studies (14–17). The strain CFBP 2526 is the pathotype strain of *Xanthomonas axonopodis* pv. glycines (18).

Both strains were sequenced using the Illumina HiSeq 2000 platform (GATC Biotech, Germany). Shotgun sequencing yielded 77,326,552 read pairs (75,568,393 100-bp paired-end reads with an insert size of 250 bp and 1,758,159 50-bp mate-pair reads with an insert size of 3 kb) and 59,188,498 read pairs (47,138,978 100-bp paired-end reads and 12,049,520 50-bp mate-pair reads) for strains CFBP 2526 and CFBP 7119, respectively. A combination of Velvet (19), SOAP*denovo*, and SOAP Gapcloser (20) yielded 25 contigs >500 bp (N_50_, 423,865 bp), with the largest contig being 1,231,354 bp, for a total assembly size of 5,250,836 bp for strain CFBP 2526, and 16 contigs >500 bp (N_50_, 1,218,836 bp), with the largest contig being 2,302,514 bp, for a total assembly size of 5,518,822 bp for strain CFBP 7119.

Both strains are fully equipped to sense and move in their environment, to protect themselves, and to acquire nutrients. The main secretion systems described in Gram-negative bacteria were present, including the type III secretion system, a major pathogenicity determinant that delivers effectors (T3Es) directly into the plant cell. At least 20 T3E genes were present in each genome. Partial *tal* sequences, which were not assembled due to their highly conserved and repeated structures, were also found. Further studies will confirm if these *tal* sequences correspond to functional genes. Most of the observed differences between the genomes correspond to plasmid sequences and to several clusters mainly featuring phage-related genes.

### Nucleotide sequence accession numbers.

These whole-genome shotgun projects have been deposited in GenBank under accession no. AUWO00000000 for strain CFBP 2526 and AUWM00000000 for strain CFBP 7119. The versions described in this paper are the first versions, AUWO01000000 and AUWM01000000, for CFBP 2526 and CFBP 7119, respectively.
